# Exercise Training-Increased *FBXO32* and *FOXO1* in a Gender-Dependent Manner in Mild Cognitively Impaired African Americans: GEMS-1 Study

**DOI:** 10.3389/fnagi.2021.641758

**Published:** 2021-04-14

**Authors:** Fikru B. Bedada, Oyonumo E. Ntekim, Evaristus O. Nwulia, Thomas V. Fungwe, Sheeba Raaj Nadarajah, Thomas O. Obisesan

**Affiliations:** ^1^Department of Clinical Laboratory Sciences, College of Nursing and Allied Health Sciences, Howard University, Washington, DC, United States; ^2^Department of Nutritional Sciences, College of Nursing and Allied Health Sciences, Howard University, Washington, DC, United States; ^3^Department of Psychiatry, Howard University Hospital, Washington, DC, United States; ^4^Department of Nursing, College of Nursing and Allied Health Sciences, Howard University, Washington, DC, United States; ^5^Division of Geriatrics, Department of Medicine, Howard University Hospital, Washington, DC, United States

**Keywords:** ubiquitin proteasome system (UPS), *FBXO32*/atrogin-1, *FoxO* transcription factor, cellular clearance system, aging, immune cells, mild cognitive impairment, neurodegeneration

## Abstract

The ubiquitin proteasome system (UPS) and *FOXOs* transcription factors play a pivotal role in cellular clearance and minimizing the accumulation of Aβ in neurodegeneration (ND). In African Americans (AAs) with mild cognitive impairment (MCI), the role of components of UPS and *FOXOs*; and whether they are amenable to exercise effects is unknown. We hypothesized that exercise can enhance cellular clearance systems during aging and ND by increasing expressions of *FBXO32* and *FOXO1*. To test this hypothesis, we used TaqMan gene expression analysis in peripheral blood (PB) to investigate the component of UPS and *FOXOs*; and provide mechanistic insight at baseline, during exercise, and in both genders. At baseline, levels of *FBXO32* were higher in women than in men. In our attempt to discern gender-specific exercise-related changes, we observed that levels of *FBXO32* increased in men but not in women. Similarly, levels of *FOXO1* increased in men only. These data suggest that a graded dose of *FBXO32* and* FOXO1* may be beneficial when PB cells carrying *FBXO32* and* FOXO1* summon into the brain in response to Alzheimer’s disease (AD) perturbation (docking station PB cells). Our observation is consistent with emerging studies that exercise allows the trafficking of blood factors. Given the significance of *FBXO32* and *FOXO1* to ND and associated muscle integrity, our findings may explain, at least in part, the benefits of exercise on memory, associated gait, and balance perturbation acknowledged to herald the emergence of MCI.

## Introduction

Early detection of Alzheimer’s diseases in an asymptomatic patient is a daunting task, as clinical symptoms develop long after preclinical pathogenic processes (Dubois et al., 2016; Wojsiat et al., 2017). The success of therapeutic methods relies on early diagnosis before the loss of neurons and dementia occur (Wojsiat et al., 2017). Conversely, the etiology of Alzheimer’s disease (AD) is linked with several manifestations that are physical, peripheral, and systemic (Morris et al., 2014). As a multifactorial disease, AD affects the CNS and peripheral systems (Morris et al., 2014), making one size fits all therapeutic strategies ineffective. These challenges call for a preventative approach, such as exercise intervention.

Evidence indicates that immune cells may play a role in AD pathogenesis (Hohsfield and Humpel, 2015; Wojsiat et al., 2017). Consequently, blood-derived cells and their infiltration (homing) into the CNS towards Aβ plaques have been proposed as therapeutic strategies against AD (Hohsfield and Humpel, 2015). For example, by eliminating Aβ deposits and preventing plaque formation, the anti-inflammatory subset of peripheral monocytes can enhance neurogenesis and produce reparative growth factors like insulin-like growth factor I (IGF-1; Gate et al., 2010; Malm et al., 2010; Schwartz and Shechter, 2010). This process is reminiscent of exercise training-induced increases in brain-derived neurotrophic factor (BDNF; Liu and Nusslock, 2018) and IGF-1 (Carro et al., 2001; Trejo et al., 2001) levels in the brain, and rejuvenate the brain, and improve memory. Further, recent reports implicate exercise to promote the trafficking of blood factors to the brain (Małkiewicz et al., 2019).

Prior studies have described the role of *FBXO32* in muscle protein regulation, protein turnover (Kriegenburg et al., 2012), muscle atrophy (Attaix et al., 2005), and autophagy (Kostin et al., 2003) in skeletal muscles and cardiac myocytes (Zeng et al., 2013). However, whether this perturbation exists at the Mild Cognitive Impairment (MCI) phenotypic stage of AD needs elucidation. Although mechanisms linking gait and balance to neurodegeneration (ND) are unknown, decreased muscle coordination and deficits are now acknowledged early clinical phenotypes of MCI and prodromal AD (Gras et al., 2015; Goyal et al., 2019). Thus, it is unclear if *FBXO32* is perturbed in older adults because of aging and pathogenesis of ND in older African Americans (AAs) with MCI; and if fitness adaptation mitigates these processes. This is important because exercise is acknowledged to improve muscle growth and quality and we have reported that Interleukin-4 (IL-4) promoted muscle fusion and growth *via* the IL-4α receptor on myoblasts (Schulze et al., 2005). The latter further suggest a link between exercise, blood factors and muscle dynamics.

The second focus was to gain insight into *FOXO1* transcription factor. *FOXO* transcription factors are considered conserved regulators of longevity (Martins et al., 2016; Hwang et al., 2018), and coordinate genes involved in cellular metabolism and resistance to oxidative stress (Webb and Brunet, 2014). *FOXOs* promote the expression of genes involved in autophagy and the Ubiquitin Proteasome System (UPS; Webb and Brunet, 2014). *FOXO* and UPS play a pivotal role in preventing the accumulation of cells with damaged DNA and cancer (Webb and Brunet, 2014; Farhan et al., 2017). Several members of the *FOXO* transcription family regulate *FBXO32* (Webb and Brunet, 2014; Dang et al., 2016). However, whether fitness adaptation exerts some of its advantageous effects through augmentation of cellular and protein clearance *via* the expression of *FBXO32* and *FOXO1* needs an investigation. Also, the concerted role of *FBXO32* and *FOXO1* in mitigating aging processes and ND has not been investigated in Peripheral Blood (PB) of AAs with MCI. Importantly, the cumulative incidence rate of neurodegenerative diseases like AD was twice among AA than Caucasians (Tang et al., 2001), while proteins and damaged organelles accumulate during neurodegenerative disorders and aging (Metaxakis et al., 2018). As age-related decline in the expression of *FOXO* could promote ND, perturbed gait, and balance (Hwang et al., 2018), we hypothesized that exercise can enhance the cellular clearance system, and mitigate much of the harm that accumulates in the cell over time during aging and ND by increasing the expression of *FBXO32* and *FOXO1*.

## Materials and Methods

The Howard University Institutional Review Board (IRB) approved the protocols used in this study. As required for studies involving human subjects, all participants completed a signed informed consent document before enrollment.

### Randomization and Blinding

By design, the study employed a single-blind controlled approach. All staff, except those who directly monitored exercise-training, were blinded to group assignments. While impractical to blind the subjects, staff who performed cognitive assessments were blinded to group assignments. Of the 42 participants who completed baseline visits, 29 participants successfully proceeded beyond randomization; 23 participants completed the 6-month visit, but RNA was successfully extracted from 21 participants as indicated in the statistics and figure legend.

### Study Participants

Study participants in this study were enrolled, as described in the GEMS-1 study (Iyalomhe et al., 2015). The sample for this analysis included 42 participants who had baseline data. Because a low level of physical activity was an inclusion criterion for the study, all participants were sedentary before enrollment. Other inclusion criteria included age ≥50 years and MCI diagnosis status (Petersen criteria; Petersen, 2004). Volunteers taking medications and or have neurological conditions that may affect memory, and those unable to exercise vigorously without causing harm to self were ineligible to participate. Detailed inclusion and exclusion criteria have been previously published (Iyalomhe et al., 2015). While we did not use the MoCA assessment tool in the study, MMSE tool was used for screening but not a part of baseline measures. We are yet to analyze or associate the expression data with their cognitive score.

Briefly, eligibility and inclusion criteria for the study subjects include age <55 years; ability to exercise vigorously without causing harm to self (Iyalomhe et al., 2015); MCI diagnostic category according to Petersen criteria (Petersen, 2004) and in good general health. Diagnosis of MCI was made using the established criteria that include memory complaints, education adjusted Mini-Mental State Examination (MMSE) scores, as described (Mungas et al., 1996; Iyalomhe et al., 2015).

### Exercise Training Protocol

Qualified and willing volunteers underwent a maximal treadmill exercise test for 3-month and 6-month using the Bruce protocol (Bruce and Hornsten, 1969) and as previously detailed (Iyalomhe et al., 2015).

### Study Sample and Processing

The sample for this analysis included those who completed baseline, 3-month, and 6-month follow-up visits and have data on the variables that informed the scientific focus of this analysis.

Fasting blood samples were collected aseptically from all eligible volunteers at baseline, 3-months, and 6-months and allowed to clot. Serum samples were separated from clotted blood and immediately kept both at −80°C until further analysis.

### Total RNA Extraction and Reverse Transcription

To assess gene expression, total RNA was extracted from clotted blood samples using Trizol-Reagent according to the manufacturer’s protocol, followed by determination of RNA concentration using the Nanodrop 1,000 Spectrophotometer (Thermo Fisher Scientific, Waltham, MA, USA). The ratio between A260/A280 was in the range of 1.75–1.95 for all samples. cDNA first strand was generated from 300 ng RNA using the High-Capacity cDNA Reverse Transcription (RT) Kit in 20 μl reaction buffer (Thermo Fisher Scientific, Waltham, MA, USA). The RT protocol consisted of 10 min at 25°C for primer annealing, 120 min at 37°C for DNA polymerization, 5 min at 85°C for enzyme deactivation, and finally cooled to 4°C indefinitely. The cDNA was stored at −20°C until further analysis.

### Gene Expression by Quantitative Real Time-Polymerase Chain Reaction (qRT-PCR)

Gene expression level was assessed using the TaqMan gene expression assay system using 2× TaqMan Fast Advanced Master Mix and 20× FAM-MGM labeled probe sets (Applied Biosystems). The 20× FAM-MGM labeled target gene-specific probes sets have the following Assay IDs: Hs01041408_m1 for *FBXO32*, Hs00231106_m1 for *FOXO1*, and Hs99999905_m1 for *GAPDH* (Thermo Fisher Scientific, Waltham, MA, USA). The TaqMan Fast Advanced Master Mix contains AmpliTaq Fast DNA Polymerase, uracil-N glycosylase (UNG), dNTPs with dUTP, ROX™ dye (passive reference), and optimized buffer components. All probe sets were used at a final concentration of 100 nM. The PCR conditions include UNG incubation for 2 min at 50°C; polymerase activation at 92°C for 10 min; 40-cycles of denaturation at 97°C for 1 s; and annealing/extension at 62°C for 20 s. Data were normalized to *GAPDH* to compensate for the efficiency of the reverse transcription and differences in the amount of input RNA. Reactions were monitored on Applied Biosystems ViiA™ 7 Real-Time PCR System, and data analyzed with corresponding ViiA7 RUO software. qRT-PCR assays were performed in duplicate on all control and experimental group and normalized against *GAPDH* and baseline control. Relative quantification of gene expression was conducted according to the 2^(−ΔΔCT)^ relative expression method as described (Livak and Schmittgen, 2001; Bedada et al., 2014).

### Gel Electrophoresis of PCR Products

To analyze reaction quality and yield of PCR product, we used the Invitrogen E-Gel Electrophoresis System and precast 2% E-Gel agarose gels with SYBR Safe and E-Gel 1 Kb Plus DNA Ladder (Thermo Fisher Scientific, Waltham, MA, USA). Gel imaging was done using Bio-Rad imager, a gel documentation system (Bio-Rad, Irvine, CA, USA).

### Statistical Methods

For baseline screening of *FBXO32* and *FOXO1*, threshold cycle (Ct) *value* of *FBXO32* amplicons for each participant at baseline was averaged and normalized to average Ct *value* of corresponding *GAPDH* and used as calibrator. Next, the Ct *value* of *FBXO32* amplicons for individual participants was normalized to the Ct *value* of the corresponding *GAPDH* and compared to calibrator value (average of Ct *value* of *FBXO32* for all participants normalized to Ct *value* of corresponding *GAPDH* at baseline). For the calculation of specific expression of *FOXO1*, a similar approach was used.

To assess the differential effect of exercise on gene expression, Ct *value* of *FBXO32* amplicons for all individual (separately for male and female) participants at baseline were averaged and normalized to average Ct *value* of corresponding *GAPDH* and were used as calibrator. Then, the average Ct *value* of *FBXO32* amplicon at the 3-month exercise time point for all male or female participants was normalized to the average Ct *value* of corresponding *GAPDH* at 3-month exercise time point and was then compared to the male or female baseline calibrator sample (average of Ct *value* of *FBXO32* for all (separately for male and female) participants normalized to Ct *value* of corresponding *GAPDH* at baseline). A similar approach was used to calculate the specific expression of *FBXO32* at 6-month in male or female participants. A similar approach was used for *FOXO1* as well.

To determine statistical significance, data were analyzed by either *t*-test or one-way ANOVA to compare intervention groups and expressed as mean ± SEM. *P* < 0.05 was considered statistically significant. For baseline subjects, *n* = 42 where, female = 27 and male = 15. For 3-month exercise time points, *n* = 15 while female = 10 and male = 5. For 6-month exercise time points *n* = 21 while female = 15 and male = 6.

## Results

### Baseline Expression of *FBXO32* and *FOXO1*

**Figure 1:**
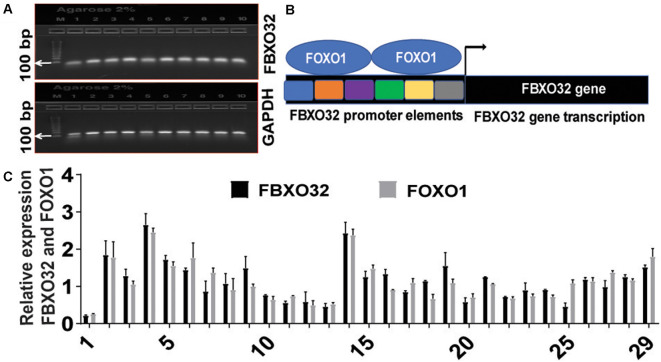
Baseline expression of *FBXO32* and *FOXO1*. **(A)** Gel electrophoresis-based detection of quantitative real time-polymerase chain reaction (qRT-PCR) products showing consistent detection of *FBXO32* and the corresponding housekeeping gene, *GAPDH*. **(B)** Schematics depicting promoter elements of *FBXO32* and the binding of *FOXO1* transcription factor on the promoter region. **(C)** Baseline quantification of *FBXO32* and *FOXO1* in real-time using the qRT-PCR. In all cases, *GAPDH* was used as an internal control and for normalization purposes. Data were run in duplicate and analyzed by one-way ANOVA and expressed as mean ± SEM, *n* = 42 for baseline subjects.

As an initial quality control check, we analyzed reaction quality, RNA integrity, cDNA first stand synthesis, and yield of PCR product using the Invitrogen E-Gel Electrophoresis System. Accordingly, gel electrophoresis-based detection of qRT-PCR products for *FBXO32* showed a consistent and specific band corresponding to *FBXO32* and *GAPDH* (Figure 1A), suggesting the cDNAs first strands were synthesized properly, and the RNAs were intact. Since *FOXO* transcription factors initiate transcription of *FBXO32* by binding to its promoter elements (Bodine and Baehr, 2014), we developed schematics depicting *FBXO32* transcription process (Figure 1B). To investigate the role of FBXO*32* and *FOXO1* in immune cells, we determined their expression levels in RNA samples isolated from clotted PB of AA with MCI. Next, we quantified *FBXO32* and *FOXO1* in real-time using the qRT-PCR. Accordingly, both genes were expressed in a cohort of 42 subjects tested at baseline (Figure 1C). In all cases, *GAPDH* was used as an internal control and for normalization purposes. Collectively, these expression data suggest that *FBXO32* and* FOXO1* might have an important role in systemic circulation and can play a therapeutic role when these PB cells carrying *FBXO32* and* FOXO1* trafficked into the brain. Specifically, recent reports implicate exercise to increase BBB permeability and promote the trafficking of blood factors to the brain (Małkiewicz et al., 2019); this view prompted us to investigate further the role of fitness adaptation on the expression level of *FBXO32* and *FOXO1* in AA with MCI.

### Gender-Dependent Expression of *FBXO32* and *FOXO1* at Baseline and Following Exercise Intervention

**Figure 2:**
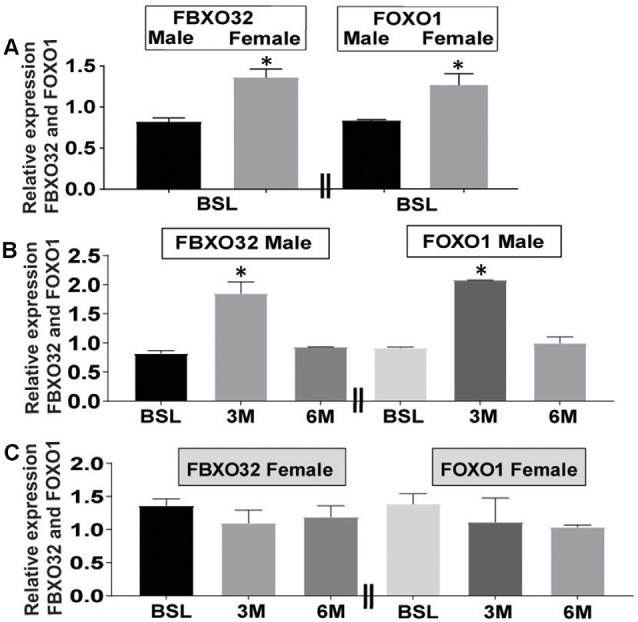
Differential expression of *FBXO32* and *FOXO1* at baseline and effect of exercise intervention by gender. **(A)** Representative data showing increased expression of *FBXO32* and *FOXO1* at baseline in female subjects (Gray bar) compared with male counterparts (Black bar). **(B)** Representative data showing increased expression of *FBXO32* in male subjects at the 3-month exercise time point (left panel) and increased expression of *FOXO1* in male subjects at the 3-month exercise time point (right panel). **(C)** Representative data showing lack of effect of exercise on the expression of *FBXO32* in female subjects at all time points (left panel) and lack of exercise effects on the expression of *FOXO1* in female subjects at all time points (right panel). Data were run in duplicate and analyzed by either *t*-test or one-way ANOVA and expressed as mean ± SEM. For baseline subjects, *n* = 42 where, female = 27 and male = 15. For 3-month exercise time points, *n* = 15 where female = 10 and male = 5. For 6-month exercise time points, *n* = 21 were female = 15 and male = 6. *P < 0.05 for panels **(A,B)**. Limitation: because the collection of 3-month blood samples was conceived after protocol initiation, this time point included fewer volunteers than the 6-month visit.

Age and gender are leading risk factors for developing AD, and female subjects tend to have a higher incidence of the disease than men (Filon et al., 2016). First, we analyzed levels of *FBOX32* and *FOXO1* at baseline in both genders. Data at baseline show that higher levels of *FBXO32* and *FOXO1* expression in female (Figure 2A and gray bar) than in male subjects (Figure 2A and black bar), suggesting females may have high reserves of *FBXO32* and *FOXO1* at baseline (Figure 2A).

Next, in our attempt to evaluate the differential effects of exercise-related changes by gender, we analyzed the level of *FBXO32* and *FOXO1* expression following exercise intervention in both genders (Figures 2B,C). Our data indicate that exercise-induced significant changes in the expression level of *FBXO32* (Figure 2B and left panel) and *FOXO1* (Figure 2B and right panel) in male subjects (Figure 2B). We evidenced an acute increase at the 3-month exercise-training time point, which dialed back to basal level following a 6-month exercise intervention (Figure 2B).

On the other hand, fitness adaptation did not induce significant changes in the expression level of *FBXO32* (Figure 2C and left panel) and *FOXO1* (Figure 2C and right panel) in female subjects (Figure 2C). Collectively, these data suggest a differential effect of gender on the expression level of *FBXO32* and *FOXO1* at baseline (Figure 2A) and following exercise intervention (Figures 2B,C).

## Discussion

The complexity of AD pathology and the challenge of early diagnosis hinder the likelihood of therapeutic success. Thus, targeting exercise intervention can be an invaluable alternative to mitigate the progression of MCI to AD. Of note, recent work establishes a link between exercise and increased permeability of BBB, facilitating the migration of therapeutic blood factors to the brain (Małkiewicz et al., 2019).

In this study, we determined if fitness adaptation can increase the expression of *FBXO32* and *FOXO1*, and therefore, potentially have favorable effects on ND, perturbed balance, and aging. Our finding has two components. First, we observed gender-based differences at baseline in the expression level of *FBXO32* and *FOXO1*. Second, we evidenced the differential effect of exercise, where a 3-month exercise intervention increased levels of *FBXO32* and *FOXO1* in males compared to females. Given the significance of *FBXO32* and *FOXO1* in ND, and muscle function, our findings, may at least in part, inform the advantageous effects of exercise on memory, declining muscle coordination, and progression of ND in AA MCI participants.Perturbed muscle coordination, gait, and balance have been suggested to herald the early phase of cognitive deterioration (Gras et al., 2015; Goyal et al., 2019). Given the role of *FBXO32* in muscle dynamics and function, and the acknowledged effects of exercise on muscle, gait and balance, we explored the effects of exercise on *FBXO32* expression. A prior study showed that IL-4, a blood factor, served as myoblast recruitment factor during muscle growth (Horsley et al., 2003). New evidence in mice demonstrates that the up-regulation of IL-4 contributes to aerobic exercise-induced insulin sensitivity (Chen et al., 2020). These observations likely suggest a link between exercise, blood factors, muscle dynamics, and metabolism. Our observation that *FBXO32* expression increased following a 3-month exercise (Figure 2B), suggests that exercise-training effects may peak at 3-month and then trend towards equilibrium at 6-month. We attribute the apparent effect at the 3-month time point to the exercise regimen. Briefly, each participant initially trained for 20 min at an intensity targeted at 50% VO2Max. We then increased training duration by 5 min each week until each participant reached 40 min of exercise at 50% VO2Max, and then increased training intensity by 5% VO2Max/week until reaching 70% VO2Max. Notably, this peaking of exercise frequency, intensity, and duration coincided with the 3-month time point. This implies that fitness level may have plateaued at 3-month, thereby motivating less changes in biomarkers. The remaining 3-month of training occurred in the context of physiologic adjustments, feedback mechanisms, and ensuring biologic adjustments. Therefore, counter regulatory mechanisms may underly the blunting, at least in part, some exercise effects at the 6-month time point. Of note, the apparent high baseline level of *FBXO32* and *FOXO1* in female subjects can potentially mount negative feedback responses to avoid further saturation of the UPS system. Therefore, female participants may not have responded to exercise intervention analogously to male participants who have a lower level of *FBXO32* and *FOXO1* at baseline.

This beneficial effect of exercise-induced increase in *FBXO32* is analogous to impaired autophagy/lysosome system in *FBXO32* deficient zebrafish (Buhler et al., 2016). Further, genetic deletion of *FBXO32* in aging rodents shortened their lifespan and caused significant muscle atrophy, corroborating the need for *FBXO32* in normal protein turnover during aging (Sandri, 2013; Sandri et al., 2013).

UPS plays an essential role in maintaining cellular quality and homeostasis (Sandri, 2013). In an experimentally induced DNA damage such as genotoxic stress, stabilization of *FBXO32* led to the activation of *NF-κB* *via* polyubiquitination and proteasome-mediated degradation of an inhibitor *IκBα* (Meshram et al., 2017). Thus, *FBXO32* is a potential activator of *NF-κB* signaling during genotoxic stress and inflammatory response (Meshram et al., 2017). Notably, AD is an inflammatory disorder; and reduced levels of nuclear *NF-κB* immunoreactivity have been observed around plaques in human AD brains (Kaltschmidt et al., 1999). Inhibition of *NF*-*κB* potentiates Aβ-mediated apoptosis in primary rat neurons (Kaltschmidt et al., 1999). *FBXO32*-mediated activation of *NF-κB* protects the cells, maintains their genomic integrity, and allow restoration of a healthy life cycle (Ryan et al., 2000; Kawahara et al., 2009; Meshram et al., 2017). Similarly, the beneficial effect of fitness adaptation through the induction of *FBXO32*, and therefore, proteolysis may counter the negative effect of *c-myc*, a proto-oncogene (Mei et al., 2015). Recently, transgenic expression of *c-myc* in neuronal cells induced significant cognitive deficits and ND (Lee et al., 2009). Since cell cycle disturbances may precede neuronal death in AD (Wojsiat et al., 2015), the exercise-induced increase in *FBXO32* in PB can counter cell cycle disturbances mitigating the progression of MCI to AD. Thus, exercise training-induced increase of *FBXO32* in our AA MCI sample may reduce inflammation, ND, and cell cycle disturbance. This is consistent with recent studies that showed a favorable effect of exercise on BBB permeability (Małkiewicz et al., 2019), facilitating the trafficking of neuroprotective blood factors to the brain (Carro et al., 2001; Trejo et al., 2001; Liu and Nusslock, 2018).

Our gender-dependent changes in *FBXO32* at baseline and after exercise (Figures 2A,B) could be linked to gender hormones such as dihydrotestosterone (DHT), estradiol (E2), and estrogen receptor (Svensson et al., 2010). For instance, reduced expression of *FBXO32* was associated with gonadectomy in male mice (Svensson et al., 2010), and treatment with DHT or E2 normalized the reduced level (Svensson et al., 2010). Gender hormones also influence FOXO expression as revealed by subcutaneous E2 implant, which improved insulin sensitivity and suppressed gluconeogenesis in both male and ovariectomized female mice (Yan et al., 2019), and insulin resistance is linked to the pathogenesis of AD (Kellar and Craft, 2020). However, these effects of E2 were abolished in *FOXO1* knockout mice, implying an interaction between *FOXO1* and E2 (Yan et al., 2019). Further, the deletion of *ERα* exhibited reduced levels of *FBXO32* and *FOXO1* (Ribas et al., 2016), underscoring the importance of gender hormones for maintaining the levels of *FBXO32* and* FOXO1*. Further, *FBXO32* RNA seq expression data from human protein atlas (HPA) data set and genotype tissue expression (GTEx) data set shows higher expression of *FBXO32* in several female tissues such as the endometrium, fallopian tube, breast, ovary, cervix uteri, placenta, and vagina compared with limited male tissues such as seminal vesicles, prostate, epididymis, and testis^1^. Similarly, higher expression of *FOXO1* is documented in several female tissues compared with male tissues^2^.

The *FOXO* transcription family members regulate *FBXO32* (Webb and Brunet, 2014; Dang et al., 2016; Yin et al., 2018). Concordant with our findings for *FOXO1*, the baseline levels of *FOXO3* mRNA were higher in older women (Raue et al., 2007), and *FBXO32* expression is also regulated by *FOXO3A* (Zheng et al., 2010; Webb and Brunet, 2014). Others have identified *FOXO* as a guardian of neuronal integrity, protecting against age-progressive axonal degeneration (Hwang et al., 2018). *FOXOs* played a role as a regulator of longevity and genes involved in cellular metabolism and resistance to oxidative stress (Webb and Brunet, 2014; Martins et al., 2016; Hwang et al., 2018). Collectively, these observations put female subjects at a unique advantage to have a large pool of *FBXO32* and* FOXO1* compared with male subjects. We contend that the apparent high level of *FBXO32* and *FOXO1* can mount negative feedback responses to avoid further saturation of the UPS system and creating imbalance. As a result, female subjects did not respond to exercise training compared with male subjects who have lower levels of *FBXO32* and *FOXO1* at baseline. We plan to further inform additional proteolytic mechanisms in conjunction with *FBXO32* following exercise intervention involving males and females in future studies.

Neurodegeneration is a complex and idiopathic disease and is an inevitable consequence of aging (Hwang et al., 2018). A prior study linked age-related decline in the expression of *FOXOs* to ND, suggesting FOXOs play a central role in neuroprotection during mammalian aging and ND (Hwang et al., 2018). Central to NDs are UPS and autophagy, two major intracellular clearing systems, the impairment of which is widely implicated in protein aggregation and neurotoxicity (Pan et al., 2008; Bedford et al., 2009; Jaeger and Wyss-Coray, 2009). Since these neurodegenerative proteinopathies operate at the crossroad between UPS and autophagy, the interplay between these degradation and clearance systems need to be balanced to ensure effective protein homeostasis (Limanaqi et al., 2020). Accordingly, recent work shows that *FOXO/FBXO32/endophilin* network may play an important role in balancing autophagy and UPS and thereby maintain neuronal health in the mammalian brain (Murdoch et al., 2016). The overall protein homeostasis and health of neurons are maintained by the interplay between *FOXO/FBXO32/endophilin* (Murdoch et al., 2016). Previously, *FBXO32* has been described as a mediator between the UPS and autophagy (Zaglia et al., 2014; Mei et al., 2015; Buhler et al., 2016; Meshram et al., 2017); and along with endophilin-A necessary for autophagosome formation in neurons and the mammalian brain (Murdoch et al., 2016). Taken together, our observations and others highlight the relationship between *FBXO32/FOXO1* and NDs.

Collectively, these studies support our observation that exercise training-induced changes in *FBXO32* and *FOXO1* expressions may, at least in part, explain the advantageous effects of exercise on neurodegenerative processes such as in MCI.

## Concluding Remarks

In this study, we evaluated the effect of fitness adaptation on gene expression in PB by investigating E3 ubiquitin ligase components of the UPS; and their regulator *FOXO1*. We demonstrate that exercise intervention increases* FBXO32* and *FOXO1* in a gender-dependent manner. However, gene expression analysis performed in this study may not capture the full spectrum of the biologic effects of exercise in AA with MCI. Further investigations that include additional genes, protein levels, and cytokines at similar time points are, therefore, required. We will inform this in future analysis.

## Data Availability Statement

The raw data supporting the conclusions of this article will be made available by the authors, without undue reservation.

## Ethics Statement

This study involved human participants and was reviewed and approved by Howard University IRB. The participants provided their written informed consent to participate in this study.

## Author Contributions

FB and TO conceived, planned, and carried out unique experiments and wrote the manuscript. ON contributed to the study, sample preparation, RNA isolation, cDNA preparation, and manuscript preparation. EN, TF, and SN contributed to the study design, study implementation, and manuscript review. TO conceived the design and implemented the overall study. All authors contributed to the article and approved the submitted version.

## Conflict of Interest

The authors declare that the research was conducted in the absence of any commercial or financial relationships that could be construed as a potential conflict of interest.

## Footnotes

^1^https://www.proteinatlas.org/ENSG00000156804-FBXO32/tissue

^2^https://www.proteinatlas.org/ENSG00000150907-FOXO1/tissue
